# Development and Application of an Optoelectronic Sensor for Flame Monitoring of a Copper Concentrate Flash Burner

**DOI:** 10.3390/s25092897

**Published:** 2025-05-03

**Authors:** Gonzalo Reyes, Walter Díaz, Carlos Toro, Eduardo Balladares, Sergio Torres, Roberto Parra, Jonathan Torres-Sanhueza, Maximiliano Roa, Carla Taramasco, Víctor Montenegro, Milen Kadiyski

**Affiliations:** 1 Metallurgical Engineering Department, Universidad de Concepción, Concepción 4070386, Chile; gonzaloreyes@udec.cl (G.R.); walterdiaz@udec.cl (W.D.); eballada@udec.cl (E.B.); rparra@udec.cl (R.P.); maxiroa@udec.cl (M.R.); 2 Faculty of Engineering, Universidad Andres Bello, Autopista Concepción-Talcahuano 7100, Talcahuano 4260000, Chile; 3 Electrical Engineering Department, Universidad de Concepción, Concepción 4070409, Chile; sertorre@udec.cl (S.T.); jonatorres@udec.cl (J.T.-S.); 4 School of Engineering, Universidad Andres Bello, Viña del Mar 2531015, Chile; carla.taramasco@unab.cl; 5 Aurubis AG, 20539 Hamburg, Germany; v.montenegro@aurubis.com; 6 Aurubis Bulgaria AD, 2070 Pirdrop, Bulgaria; m.kadiyski@aurubis.com

**Keywords:** optical sensors, spectral radiation, flash furnace, flame characterization, optoelectronics, spectroscopy, copper concentrate, flash oxidation, sulfides

## Abstract

**Highlights:**

**What are the main findings?**

- The developed optoelectronic prototype, using a VIS-NIR spectrometer and high-temperature optical fiber, successfully captured key combustion parameters—including total radiation, flame temperature, and flickering dynamics—under real industrial conditions in a copper flash smelting furnace.
- Fourier-based analysis, including STFT, revealed intrinsic low-frequency oscillations and instability events, clearly characterizing the pulsating behavior and combustion anomalies of the FSF burner.

**What is the implication of the main finding?**

- The integrated sensing system offers a robust, non-invasive, and real-time diagnostic tool that enhances the operational control and efficiency of copper pyrometallurgy.
- Detailed insights into flame dynamics provided by the sensor will pave the way for the development of automated control strategies and predictive maintenance in industrial smelting operations.

**Abstract:**

A flash smelting furnace operation is based on the exothermic reduction of copper concentrates in the combustion shaft, and these reactions occur at high temperatures (1250–1350 °C), where flame control is fundamental to optimizing copper reduction. Furthermore, inherent physicochemical reactions of the reduction process have been shown to emit spectral lines in the visible-near infrared spectrum (250–900 nm). Thus, an optoelectronic sensor prototype is proposed and developed for flame measurements of an industrial copper concentrate flash smelting furnace. The sensor system is composed of a high-temperature optical fiber probe, which functions as a waveguide to capture the emitted flame radiation and a visible-near infrared spectrometer. From the measured radiation, flame temperature and flame dynamics are analyzed. Flame temperature is estimated using the two-wavelength temperature estimation method, and flame dynamics are defined as variations in the total emissive power, which are studied in the time and frequency domain via the Fourier Transform method. These combustion dynamics are then used to create a flame instability index, which is used to characterize the flame combustion quality. The combination of this index and sensor platform provides a powerful tool to aid in proper flame control.

## 1. Introduction

Modern industry implements a wide range of sensors and intelligent control systems that effectively support decision-making, enabling maximum efficiency through high levels of automation in industrial plants. Although many industrial examples can be found, the petroleum and steel industries pioneered the adoption of the fourth industrial revolution in their control systems [1,2,3,4,5,6,7,8,9,10,11,12,13].

In contrast, the implementation of sensors and intelligent control systems remains limited or nonexistent for copper production pyrometallurgical processes, giving the human factor a fundamental role in decision-making and control strategies during the smelting and conversion stages of copper extraction processes [1]. In this work, we propose an optoelectronic integrated sensing and analysis system to solve the lack of digital feedback information from these processes.

The steel Industry, featuring processes like those used In the copper Industry, Implements diverse sensors and well-developed control systems that are vital for operational flexibility. This approach enables a comprehensive analysis of productivity, quality standards, key parameter indexes, and overall process efficiency.

These conditions show a clear difference between the two industries, with the copper industry operating at a comparatively lower level of technological maturity than the steel industry. For instance, the copper industry’s specific refractory consumption is at least five times higher, whereas steel operations use advanced predictive models that minimize human dependence and achieve a more controlled operation.

In the specific context of extracting information from the spectral radiation produced by high-temperature systems, the steel industry has demonstrated that radiometric techniques can discriminate steel from slag during oxygen converter tapping, enabling spatial separation of both phases [2,3,4,7].

This type of sensor has been successfully deployed in industrial facilities since the 1970s [5,14] and achieved maturity in the late 1980s [5,15]. In contrast, copper smelters have limited access to optoelectronic sensors, relying predominantly on static mass and energy balances for operational control.

To date, the only optical instruments that have achieved a level of success in the pyrometallurgical copper industry have been developed by Boliden Mineral AB and Noranda Inc. The former introduced the Semtech Online Production Control (OPC) system, which provides continuous information on the status of the conversion process in Peirce Smith reactors [16].

The instrument uses spectral measurements to detect the presence of PbS and PbO in the converter gas outlet. Noranda Inc. developed the Noranda pyrometer, specifically designed for smelting and converting systems using classical blowing tuyeres [16]. The device implements a two-wavelength pyrometry method to monitor the bath temperature. This instrument has been on the market since 1980, and it has remained unchanged from its original design.

Researchers at the University of Concepción have conducted extensive studies on the combustion spectra generated by copper concentrates oxidation [1,17,18]. Importantly, Parra et al. [17] demonstrated a strong relationship between the emitted spectrum and the chemical and mineralogical properties of these concentrates using an optoelectronic system under laboratory flash smelting conditions in a Drop Tube furnace.

Generally, the spectrum aligns closely with the underlying physicochemical processes when examining its baseline and discontinuous spectral features separately. Simultaneously, the classical application of Planck’s Law enables the measurement of the flame temperature resulting from the combustion of industrial copper concentrates.

This paper describes the testing of an optoelectronic system under industrial conditions in the copper Flash Smelting Furnace (FSF) at the Aurubis Smelter in Pirdop, Bulgaria, and is organized as follows: Section 2 outlines the experimental methods, including the design of the optoelectronic system, its installation in an industrial FSF, and the data acquisition procedure; Section 3 presents the results, with subsections detailing total radiation measurements, flame temperature analysis, and Fourier-based flame flickering characterization; Finally, Section 4 outlines some conclusions with key findings and the potential for future implementation of automated control strategies in copper pyrometallurgical operations.

## 2. Materials and Methods

### 2.1. Industrial Measurements Setup

The flash furnace burner is the main element of the FSF for copper concentrates, facilitating combustion by mixing dry copper concentrate feed, fluxes, circulating material, metallurgical flue dust, and oxygen-enriched air to produce fast-forming, highly exothermic oxidation reactions.

**Figure 1:**
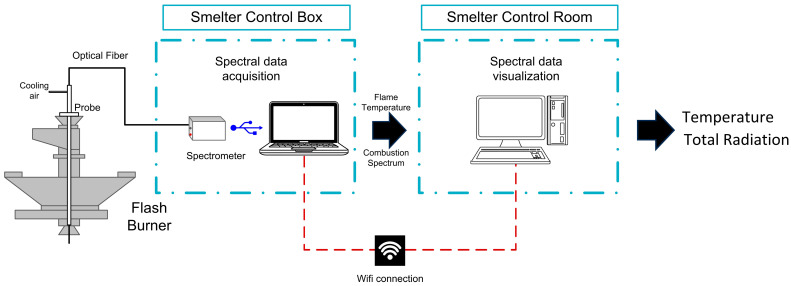
Schematic of the optoelectronic system installed at Aurubis Smelter, Bulgaria.

Continuous monitoring of the burner in a flash smelting furnace (FSF) is critical for effective process control. To address this challenge, a high-temperature fiber optic and spectrometer system was installed at the center of the Aurubis Bulgaria FSF burner in Pirdrop, Bulgaria, as shown in Figure 1.

A spectrometer (AvaSpec-ULS2048) and an optical fiber from Avantes Inc.^®^ (Louisville, CO, USA), capable of measuring in the visible-near infrared (VIS-NIR) spectral range, were used. The spectrometer digitizes the spectral radiation within a range from 200 nm to 1100 nm, with a total of 2048 discrete wavelength samples. The optical fiber operates at temperatures up to 300 °C and consists of a bundle of seven individual fibers, each with a core diameter of 400 µm. Since the spectrometer is a low-temperature device (operational temperature of 30 °C), it is housed in a protective box with an active cooling system to maintain stable operation.

**Figure 2:**
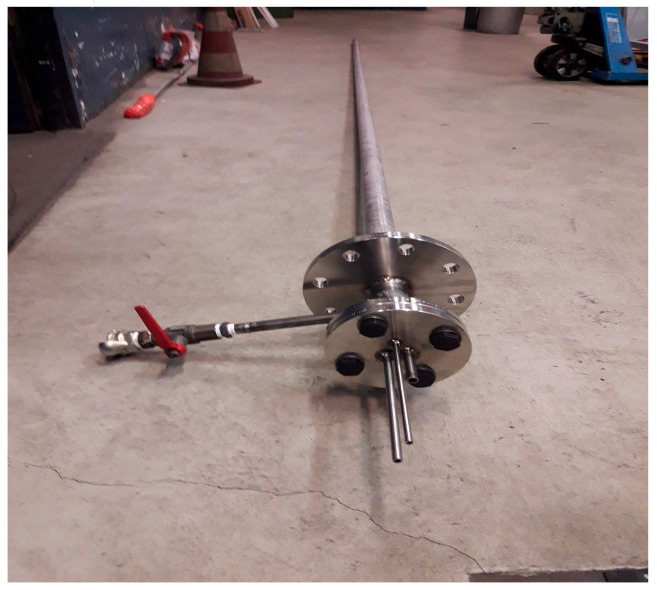
Protective housing for the optical fiber.

Even though the optical fiber has a high temperature of operation, to ensure functionality at an oven that reaches temperatures over 1000 °C, a custom-made housing equipped with an air-cooling system (Figure 2) was designed.

**Figure 3:**
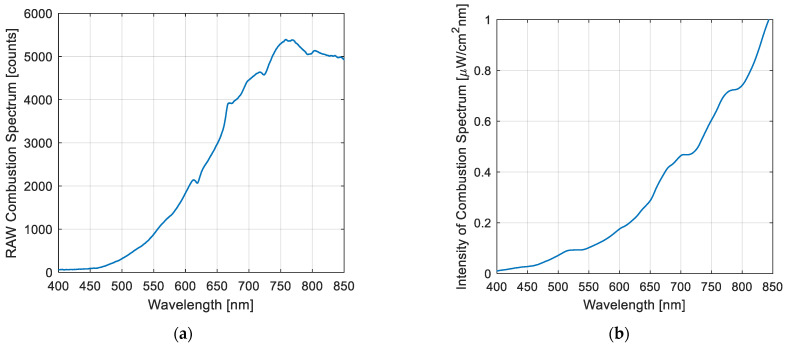
Combustion spectrum of copper concentrates: (**a**) raw spectrum; (**b**) calibrated spectrum.

Radiation was continuously captured and visualized using software developed with both MATLAB^®^ (The MathWorks, Inc., Natick, MA, USA) and LabVIEW^®^ (National Instruments Corporation, Austin, TX, USA). Over a continuous two-month period, the optical system acquired and quantified two key variables: (1) total radiation, which is related to luminosity, flame dynamics, and combustion quality, and (2) flame temperature. Both parameters were estimated from the baseline spectra, consistent with previous studies [1]. Prior to installation, the optical circuit was calibrated to correct for any electro-optical interference that the probe might receive. For this purpose, an HL-2000 CAL light source (OceanOptics^®^, Dunedin, FL, USA) was used. This calibration allowed the transformation of the digital counts into optical power units (µW/cm^2^·nm). Figure 3 shows both the raw spectrum (left) and calibrated spectrum (right), closely matching the spectra obtained in laboratory-scale tests [17].

The calibrated spectrum, Figure 3b, is a signal that can be used to describe flame energy, flame dynamics (via total radiation), and the relationship between these radiometric parameters and flame stability. In addition, the combustion spectrum was used to calculate and evaluate the flame temperature.

The calibration method applies an equation that relates the raw spectrum, integration time, and calibration vector to the characteristics of the optical probe [18]. Finally, like hydrocarbon flames, a Fourier Transform approach was applied to assess and quantify flame flickering in a copper concentrate burner [9,10,19,20].

The measurement system demonstrated robust performance under industrial conditions, continuously capturing high-intensity signals over multiple days at an acquisition rate of approximately three spectra/s achieved with an average integration time of approximately 300 ms. This parameter ensured high signal-to-noise ratios.

### 2.2. Radiometric Model

Before doing any analysis, it is important to describe the physical model used in this work to describe the measured radiation. It is known that every physical body continuously emits electromagnetic radiation. For thermal radiation, this spectral density can be described as follows:

(1) $$I\left(\lambda ,T\right)=\epsilon \left(\lambda ,T\right)\cdot I_{BB}\left(\lambda ,T\right),$$

where $I\left(\lambda ,T\right)$ is the body’s spectral density as a function of wavelength $\left(\lambda \right)$ and temperature $\left(T\right)$, $I_{BB}\left(\lambda ,T\right)$ is the spectral density of a perfect emitter, also known as a black body, and $\epsilon \left(\lambda ,T\right)$ is the emissivity of the measured object.

The spectral density of a black body is described by Planck’s law (Equation (2)) and is known as the maximum possible energy a physical body can emit at a predetermined temperature and wavelength.

(2) $$I_{BB}\left(\lambda ,T\right)=\frac{2hc_{0}^{2}}{\lambda^{5}\left[\exp\left(\frac{hc_{0}}{\lambda kT}\right)-1\right]},$$

Here, *h* and *k* are Planck’s and Boltzmann’s constants, respectively. The use of emissivity in the description of the spectral density stems from the fact that no practical body measured has the radiation energy of a black body. Therefore, emissivity is used to describe the ratio of power emitted from a radiative body compared to that of a perfect emitter. This formulation is seen in Equation (3):

(3) $$\epsilon \left(\lambda ,T\right)=\frac{I\left(\lambda ,T\right)}{I_{BB}\left(\lambda ,T\right)},$$

To simplify most of the analysis conducted in our work, we consider the measured radiation ($I\left(\lambda ,T\right)$) and the objects’ emissivity ($\epsilon \left(\lambda ,T\right)$) to be independent of direction, i.e., our measured object is a diffuse emitter.

The total amount of radiation emitted is also of particular interest to our work. The total emitted radiation of a body is the rate at which radiation is emitted per unit area at all possible wavelengths and in all directions. This is conducted by integrating over the spectrum as follows:

(4) $$I_{tot}\left(T\right)=\int_{400\;nm}^{850\;nm}I\left(\lambda ,T\right)d\lambda ,$$

In this work, total radiation is calculated over the spectral range from 400 nm to 850 nm, ensuring high signal-to-noise ratios.

### 2.3. Temperature Estimation

From the measured radiation, if knowledge of the physical bodies’ emissivity is known, an estimation of the object’s temperature can be conducted. This is known as the two-color pyrometry method or two-wavelength temperature estimation. For this temperature estimation method to be valid with our measured object, the following inequation must be true:

(5) $$\lambda T\ll \frac{hc}{k},$$

If this condition is satisfied, the measured object’s temperature can be estimated as:

(6) $$T=\frac{\frac{hc}{k}\left(\frac{1}{\lambda_{2}}-\frac{1}{\lambda_{1}}\right)}{\ln\left(\frac{I\left(\lambda_{1},T\right)}{I\left(\lambda_{2},T\right)}\right)+\ln\left(\frac{\epsilon \left(\lambda_{2},T\right)}{\epsilon \left(\lambda_{1},T\right)}\right)-5\ln\left(\frac{\lambda_{1}}{\lambda_{2}}\right)},$$

Since precise knowledge of the emissivity of an object is difficult for complex bodies like the one of a flash furnace flame. The choice of wavelengths $\lambda_{1}$ and $\lambda_{2}$ must be conducted in such a manner that our measured object acts as a gray body, i.e., the emissivity is constant throughout this chosen bandwidth. In this work, the chosen wavelengths to achieve this were 650 nm and 750 nm. By doing so, Equation (6) can be simplified to:

(7) $$T=\frac{\frac{hc}{k}\left(\frac{1}{\lambda_{2}}-\frac{1}{\lambda_{1}}\right)}{\ln\left(\frac{I\left(\lambda_{1},T\right)}{I\left(\lambda_{2},T\right)}\right)-5\ln\left(\frac{\lambda_{1}}{\lambda_{2}}\right)},$$

Thus, the accuracy of our temperature measurements will only depend on the signal-to-noise ratio of our measured radiation.

## 3. Results and Discussion

### 3.1. Total Radiation Analysis

**Figure 4:**
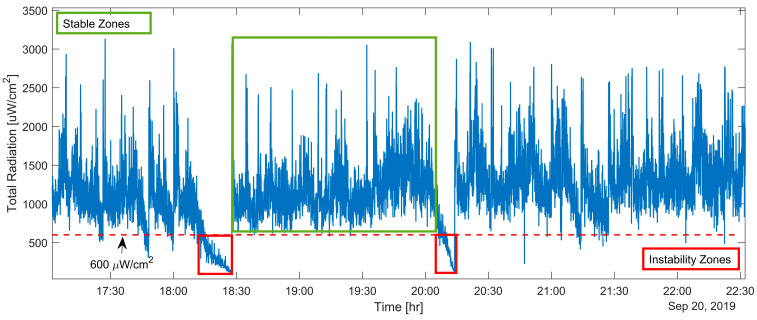
The behavior of total radiation emitted by the copper concentrate flame.

The total radiation emitted by the flame was measured continuously over two months in an industrial flash furnace operation. This parameter exhibited periodic fluctuations between pronounced peaks and valleys, potentially reflecting the intrinsic flame behavior or operational causes. Figure 4 shows an example of these observations from the measurement campaign conducted on 20 September 2019. In this figure, the total measured radiation from the flame follows a periodic pattern: it drops to low-energy levels before rapidly increasing and emitting higher energy.

Additionally, the total radiation signal in Figure 4 was segmented into stable and unstable zones using a threshold value of 600 µW/cm^2^ (red dotted line). In the stable zone (total radiation ≥ 600 µW/cm^2^), the flame temperature exhibited significantly higher values, with a mean of 1240.1 °C and a broad distribution (standard deviation = 372.7 °C), reaching a maximum of 3133.0 °C and a minimum of 600.3 °C. This widespread reflects the natural variability of combustion under normal operating conditions while maintaining overall energy release within an expected range.

In contrast, the unstable zone (total radiation < 600 µW/cm^2^) showed markedly lower temperature values, with a mean of 351.4 °C, a standard deviation of 147.8 °C, and a maximum temperature of only 598.1 °C. These values are well below the normal combustion range, confirming the absence or severe degradation of flame presence during these intervals.

**Figure 5:**
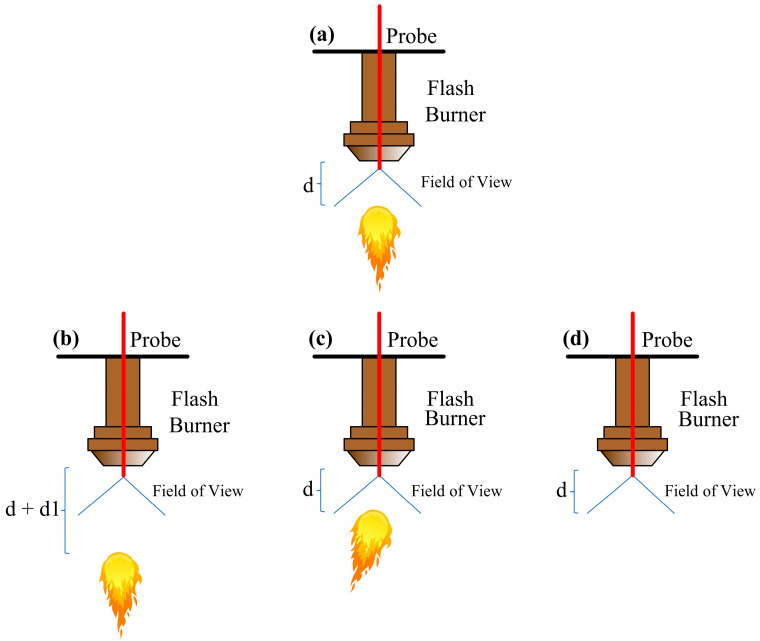
Flame behavior: (**a**) optimum condition; (**b**) flame formation far from the burner border; (**c**) deflected flame; and (**d**) no flame.

Moreover, fluctuations shown in Figure 4 are linked to specific combustion conditions, such as (1) flame development far from the concentrate discharge at the burner’s edge, (2) downward flame deviation from the field of view of the sensor, or (3) combustion interruption (flame extinguishing), as shown schematically in Figure 5. By relating the radiometric measurements with process variables over time, it can be confirmed that these signal variations are produced from combustion events rather than possible furnace stoppages. Such occurrences are typically associated with suboptimal combustion, which produces excessive dust and incomplete concentrate particle reactions. Therefore, detecting these events on time is crucial, as it allows operators to implement preventive actions and return the process to normal operations.

An extensive understanding and control of flame behavior can significantly improve operational and combustion strategies. To achieve this goal, we propose an instability index derived from the total radiation measurements for combustion monitoring.

The proposed index quantifies flame instability by measuring how long the flame remains stable—i.e., when the total radiation exceeds a specified reference threshold (600 µW/cm^2^ in this work) and identifying intervals in which the total radiation falls below this threshold, indicating an instability event.

**Figure 6:**
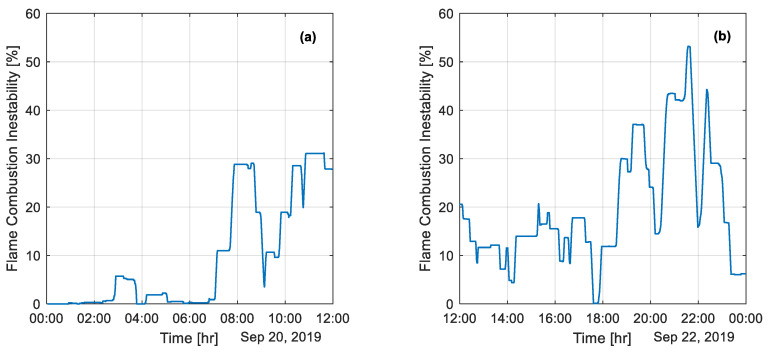
Flame instability index during two measured periods: (**a**) a 12-hour measurement period on 20 September 2019; (**b**) a 12-hour measurement period on 22 September 2019.

Figure 6 illustrates two extreme flame conditions in the flash burner. In Figure 6a, from 0 h to 2 h, there were no instability events, and the flame remained stable with continuous combustion, showing minimal energy fluctuations and reaching up to 30% instability. In contrast, Figure 6b reveals that between 21 and 22 h, the instability index exceeded 40%, reflecting numerous low-signal events that significantly influenced flame dynamics and behavior.

### 3.2. Temperature Analysis

Measuring the flame temperature in a copper concentrate burner is challenging owing to technical and mechanical constraints under operational conditions. Nonetheless, fluid dynamic models examining the combustion cloud in a flash burner have successfully assessed the thermal behavior of flash-melting furnaces [21]. In this study, two-color pyrometry was used to measure flame temperature [22,23,24].

**Figure 7:**
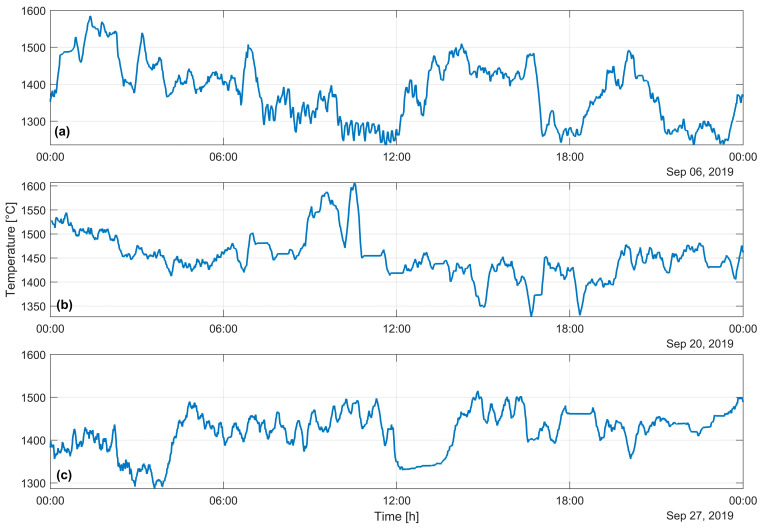
Temperature dynamic of the flame in a copper concentrate flash oxidation for three different days, statistical summary for each signal: (**a**) *T*_mean_ = 1377.8 °C, σ_T_ = 79.6 °C, *T*_min_ = 1235.5 °C, *T*_max_ = 1584.8 °C, (**b**) *T*_mean_ = 1453.6 °C, σ_T_ = 46.5 °C, *T*_min_ = 1327.6 °C, *T*_max_ = 1607.0 °C, (**c**) *T*_mean_ = 1420.5 °C, σ_T_ = 46.1 °C, *T*_min_ = 1286.5 °C, *T*_max_ = 1515.4 °C.

The temperature exhibited dynamic behavior, ranging from approximately 1300 to 1600 °C, based on operational factors such as feed composition and oxygen enrichment. These findings are consistent with those reported in laboratory studies [1,17]. Figure 7 illustrates the temperature behavior. The fluctuating temperature behavior is aligned with the expected operational range and according to CFD models of flash combustion [25]. Certain intervals exhibited abrupt drops of approximately 100 °C, while the others displayed minor oscillations.

All mean temperatures fall within a relatively narrow range, between approximately 1370 °C and 1450 °C, indicating that the general thermal conditions remain within expected operational limits. Additionally, the temperature fluctuations in all cases are centered around similar values, with a mean standard deviation of 57.4 °C, and no profile shows long-lasting deviations outside the typical process window. This straightforward analysis suggests that operator-driven temperature control can be tracked using an optical sensor.

### 3.3. Fourier Transform Analysis: Flame Flickering

In industrial processes that use hydrocarbon burners, examining the flame’s chemical properties is a standard practice for accurate process characterization. Previous studies [9,10,11,12] have relied on spectral data to control the variables in hydrocarbon burners, primarily focusing on flame flickering because it is directly associated with combustion characteristics. Investigating the oscillation frequency can reveal the stability conditions, making flickering measurements essential for understanding the key phenomena in a copper concentrate burner. In this study, flame flickering was assessed using the total radiation signal of a copper concentrate combustion flame.

To identify the frequency components, the Fourier Transform (FT) was applied to convert the time-domain signal *f*(*t*) into its frequency-domain representation *f*(*ω*). In this study, the FT was applied to the total radiation signal in the following form:

(8) $$f\left(\omega \right)=\int_{-\infty }^{\infty }f\left(t\right)e^{-i\omega t}dt$$

Flame flickering was estimated from the power spectral density (PSD). The PSD indicates the energy distribution across the frequency components of the signal. Thus, the flickering (F) in a copper concentrate flame follows the same definition used for hydrocarbon flames:

(9) $$F=\frac{\sum_{i=1}^{n}p_{i}f_{i}}{\sum_{i=1}^{n}f_{i}}$$

where:

*p_i_*: power spectral density at the *i*-th frequency component.
*f_i_*: oscillation frequency at the *i*-th frequency component.

FT was applied to continuous segments of the total radiation signal, selected from a time window free of interruptions caused by spectrometer saturation.

**Figure 8:**
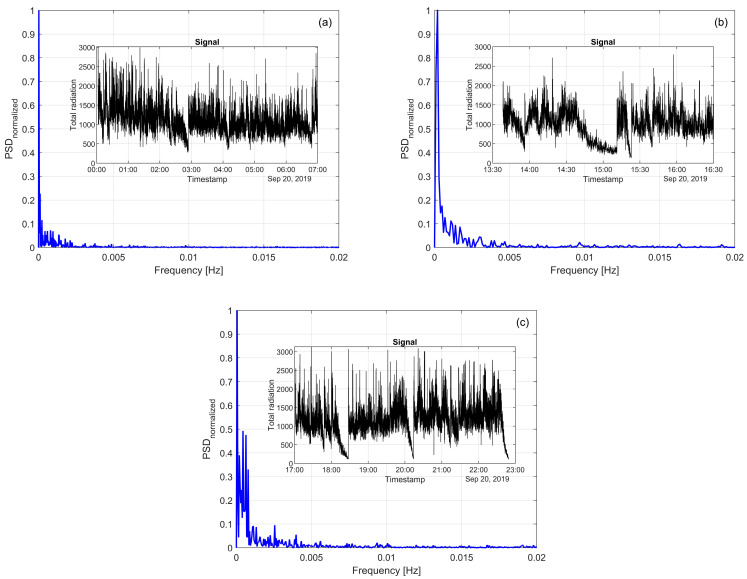
Normalized power spectral density for total radiation emitted by the flame in a copper concentrate flash oxidation during three periods of time on 20 September 2019: (**a**) a 7-hour measurement period; (**b**) a 3-hour measurement period; (**c**) a 6-hour measurement period.

Figure 8 shows the normalized spectral power density (blue lines) of the flame’s total radiation signals, with the original signals displayed in the smaller black-lined subplots, measured over uninterrupted intervals of 7, 3.5, and 6 h (Figure 8a, Figure 8b, and Figure 8c, respectively). Distinct behavior is observed at frequencies below 1 Hz.

Previous studies on hydrocarbon flames have attributed such low-frequency components to geometric fluctuations resulting from aerodynamic or convective effects, while high-frequency components reflect energetic transitions of radicals or changes in the energy release rate [9,12]. Although the overall radiation intensity remains relatively constant, minor variations in the signal’s slope suggest that the flame occasionally deviates from the optical path of the probe or that fewer particles combust in its vicinity. Such behavior can be attributed to physical phenomena commonly observed in turbulent combustion processes. In flash smelting systems, similar mechanisms are plausible, as the interaction between oxygen-enriched air and the feed of fine copper concentrate particles creates complex, time-dependent flow structures. These can induce periodic displacements or pulsations in the flame shape and position. When the flame partially shifts out of the optical path, or when the combustion zone becomes locally diluted, the emitted radiation reaching the probe is reduced, leading to low-energy events in the signal. These structural and positional flame changes can manifest in the frequency domain as persistent low-frequency components during stable conditions.

**Figure 9:**
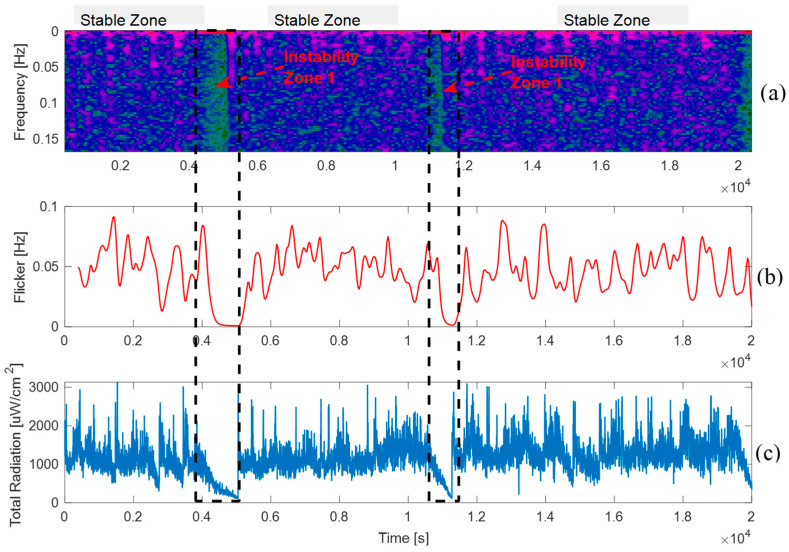
Flickering and frequency analysis of a copper concentrate flame: (**a**) Spectrogram; (**b**) Flickering dynamic; (**c**) Total radiation.

Flame flickering was calculated from the frequency representation of total radiation using Equation (9). Additionally, a Short-Time Fourier Transform (STFT), derived from the FT outlined in Equation (8), was implemented to process equal-sized time windows of the temporal signal separately. Once the stable and unstable zones of the signal were identified, STFT was applied to determine the differences in the frequency components and energy transitions between these regions. The temporal evolution of the flickering signal was also calculated. The results are shown in Figure 9.

The spectrogram in Figure 9a shows the energy levels for every frequency component of the analyzed signal over time, representing the time and frequency. Higher energy values are shown in purple, and small or zero energy values are shown in green. Within the analyzed time interval, a persistent low-frequency component is evident. The stability zone is composed of sinusoidal signals ranging from 0 to 0.15 Hz, where low energy signals appear and disappear periodically over short periods of time. When analyzing the red-pointed zones, we can observe the signal begins to decrease and enters an instability zone, which translates into a low-frequency signal. That is, energy is observed sporadically. Thus, the components that define a stable signal (stable zone) completely disappear. This behavior indicates that, at this point, the flame is undergoing a poor combustion event, as the measured radiation is nearly absent.

Figure 9b shows the flickering dynamics in the stable zone, which reveals a pulsating and oscillatory behavior of the flame. The flame fluctuations accelerate and decelerate within a period of approximately 1000 s, indicating an intrinsic oscillation condition. However, in the unstable zone, the oscillatory characteristics disappear owing to signal decay, and the flickering approaches zero. This suggests that the probe is not detecting the flame, likely because of significant deflection and/or episodes of non-ignition. Note that the analyses presented in Figure 8 and Figure 9 were conducted under continuous and stable industrial operating conditions, and they accurately describe the flame behavior in an FSF burner. Moreover, to complement this analysis, it is important to mention that the spectral resolution is inherently limited by the system’s acquisition rate of one spectrum every three seconds. According to the Nyquist sampling theorem, this restricts the analysis to phenomena below 0.166 Hz. As such, the observed frequency components reflect only slow oscillatory behavior and may not capture higher-frequency dynamics such as typical flame flickering, which is often reported above 10 Hz in hydrocarbon combustion studies. This constraint, if unacknowledged, may lead to aliasing or misinterpretation. Therefore, while the current analysis provides valid insights into low-frequency instability patterns, future studies should aim to increase the sampling frequency to enable a more precise and comprehensive characterization of the flicker phenomenon.

### 3.4. Correlation Analysis

**Figure 10:**
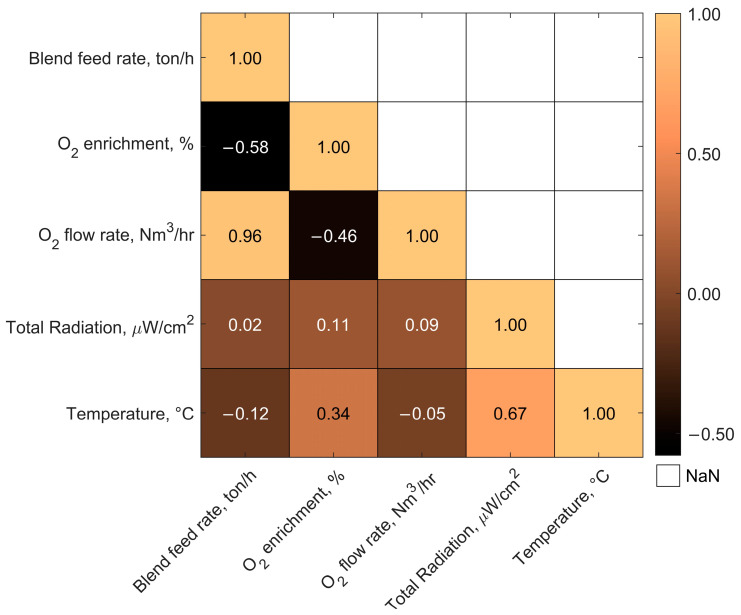
Correlation analysis between operational variables and radiometric estimated variables with data from 20 September 2019.

Finally, Figure 10 shows a correlation analysis between operational variables and radiometric estimated values. In this analysis, a correlation matrix between the blended concentrates feed rate, oxygen enrichment, oxygen flow rate, total radiation, and estimated temperature is depicted.

A strong positive correlation (0.96) is observed between the blend feed rate and the oxygen flow rate, indicating a consistent adjustment of oxygen supply with increasing material input. In contrast, oxygen enrichment shows a moderate negative correlation with both feed rate (−0.58) and oxygen flow (−0.46), suggesting that enrichment is reduced when either the total flow or feed increases, possibly to control combustion intensity or temperature profiles.

Interestingly, the total radiation exhibits weak correlations with operational parameters, with the highest being a slight positive correlation with oxygen enrichment (0.11), while calculated flame temperature shows a moderate correlation with oxygen enrichment (0.34) and a strong positive correlation with total radiation (0.67). These results confirm the consistency of radiometric temperature estimation with the emitted energy of the flame and highlight the relevance of enrichment as a factor influencing combustion temperature despite its lower influence on total radiation levels.

### 3.5. Practical Considerations

In addition to its monitoring capabilities, the sensor system requires careful attention during installation and maintenance to ensure optimal performance under harsh industrial conditions.

During installation: (i) The optical fiber probe was positioned axially along the flame direction, aligned with the main combustion axis, to maximize the captured radiation intensity and ensure optimal signal collection from the core reaction zone. (ii) A stable mechanical interface with vibration damping is recommended to reduce any optomechanical connection failure. (iii) The cooling system must provide a continuous airflow through the protective housing to prevent fiber degradation. The airflow should be of instrumentation-grade quality, incorporating filtering stages to remove particulates and moisture.

Moreover, during the two-month measurement campaign described in this work, the system operated continuously without requiring any maintenance, demonstrating its robustness and reliability under industrial conditions. Nevertheless, for long-term deployment or in environments with different operational dynamics, we recommend the following preventive maintenance practices to ensure continued performance: (i) Calibration using a known light source (e.g., HL-2000 CAL) is advisable following any planned burner shutdown or in case of suspected optomechanical disturbances, to verify the stability and accuracy of the measurements, (ii) The optical fiber tip should be inspected and cleaned if a persistent decrease in signal-to-noise ratio or radiation amplitude is observed, which may indicate dust accumulation or partial optical obstruction.

## 4. Conclusions

The optical measurement prototype has proven to be a robust tool under a wide range of extreme industrial conditions. It enables real-time temperature and total radiation monitoring of an FSF burner, even in environments with high dust concentrations, elevated temperatures, and constant mechanical vibrations. The optoelectronic system not only allowed for the identification and detailed study of the dynamic flame behavior in a flash burner, but it also facilitated the estimation of flame temperature and the detection of instability events inherent to the physicochemical process.

The measured signal, predominantly composed of low-energy sinusoidal components, suggests that the probe may receive reduced radiation during instances of flame deflection or non-ignition. Moreover, the system effectively captured pulsating and oscillatory dynamics, as evidenced by both the Fourier and flicker spectra.

Fourier analysis revealed that the flame primarily exhibits low-frequency oscillations, likely caused by slow physical movements that shift the flame out of the sensor’s field of view. The observed flickering, with a period of approximately 1000 s, indicates periodic acceleration and deceleration of the flame dynamics. Although these preliminary findings are subject to the current sampling frequency, they highlight the potential of this integrated hardware–software system as a non-invasive, robust, and easily deployable sensor for real-time diagnostics and operator decision support in copper flash smelting operations.

In future work, multiple-point measurements will be conducted to enable spatially resolved flame monitoring and advanced combustion mapping. Additionally, machine learning models to correlate flame features (e.g., flickering signatures) with specific operational states or faults (e.g., feed anomalies, incomplete combustion) or for prediction of critical output variables (e.g., copper content or slag composition) will also be developed.

## Data Availability

The datasets presented in this article are not readily available because they include proprietary industrial data subject to confidentiality agreements and are also part of a broader ongoing research project. Requests to access the datasets should be directed to Gonzalo Reyes, gonzaloreyes@udec.cl.

## Abbreviations

The following abbreviations are used in this manuscript:

VIS	Visible
NIR	Near Infrared
FSF	Flash Smelting Furnace
FT	Fourier Transform
STFT	Short Time Fourier Transform
OPC	Online Production Control
PSD	Power Spectral Density

## References

[1] Arias L., Balladares E., Parra R., Sbarbaro D., Torres S. (2020). Sensors and Process Control in Copper Smelters: A Review of Current Systems and Some Opportunities. Minerals.

[2] Viale M., Martin O., Muratori F., Bertezzolo U., Perez J., Usart J. Application of On-Line Infrared Thermography in Steel Making Industry. Proceedings of the Thermosense XXIX.

[3] Wittchen W., Borecki M., Więcek B., Pacholski K., Olbrycht R., Strąkowski R. (2015). Multispectral Measuring System for Determination of Steel Slag Parameters during Steel Tapping from Metallurgical Furnace to Casting Ladle. Prace Instytutu Metalurgii Żelaza.

[4] Więcek B., Pacholski K., Świątczak T., Wittchen W., Borecki M. Multispectral System for Measuring the Radiation Parameters of Steel Slag during the Discharge of Steelworks Furnace. Proceedings of the 11th International Conference on Quantitative Infrared Thermography.

[5] Chiba K., Ono A., Saeki M., Ohno T., Masao Y., Kanamoto M. (1991). On-Line Analysis of Molten Steel in Converter. Anal. Sci..

[6] Ball D.F., Bullock J.D., Filer D.P., Samain M.D., Shewring D., Watts C. Emission Spectra from Linz-Donawitz Converters. Proceedings of the Symposium on Process Instrumentation in the Metals Industry.

[7] Strąkowski R., Pacholski K., Więcek B., Olbrycht R., Wittchen W., Borecki M. (2014). Radiative Parameters of Steel Slag for FeO Content Estimation Using Multispectral Thermography System. Quant. InfraRed Thermogr. J..

[8] Zhang Z., Bin L., Jiang Y. (2014). Slag Detection System Based on Infrared Temperature Measurement. Optik.

[9] Hamins A., Yang J.C., Kashiwagi T. (1992). An Experimental Investigation of the Pulsation Frequency of Flames. Symp. Int. Combust..

[10] Cetegen B.M., Ahmed T.A. (1993). Experiments on the Periodic Instability of Buoyant Plumes and Pool Fires. Combust. Flame.

[11] Weiser V., Eisenreich N. (2005). Fast Emission Spectroscopy for a Better Understanding of Pyrotechnic Combustion Behavior. Propellants Explos. Pyrotech..

[12] Huang Y., Yan Y., Lu G., Reed A. (1999). On-Line Flicker Measurement of Gaseous Flames by Image Processing and Spectral Analysis. Meas. Sci. Technol..

[13] Ball D.F., Bullock J.D., Filer D.P., Orville-Thomas W.J., Samain M.D., Shewring D., Watts C. Radiation Measurements for the Control of LD Steelmaking. Proceedings of the International Conference and Exhibition on Industrial Measurement and Control by Radiation Techniques.

[14] Chiba K., Ono A., Ohno T., Okajima M., Yamane H., Hayata M. (1987). Direct Measurement of Manganese in Molten Iron with Observing Spectrum from Hot-Spot. Trans. Iron Steel Inst. Jpn..

[15] Diaz C., Levac C., Mackey P., Marcuson S., Schonewille R., Themelis N., Kapusta J., Mckey P., Stubina N. (2011). Innovation in Nonferrous Pyrometallurgy—1961–2011. The Canadian Metallurgical & Materials Landscape 1960 to 2011.

[16] Prietl T., Filzwieser A., Wallner S. Productivity Increase in a Peirce-Smith Converter Using the COP KIN and OPC System. Proceedings of the 134th TMS Annual Meeting.

[17] Parra R.A., Parra V.R., Balladares E.R., Loeza C.A., Villagrán C.M., Pérez M., Torres S.N., Arias L.E., Sbárbaro D. Online Temperature Measurements during Copper Concentrate Flash Combustion at Laboratory Scale by a Spectral Technique. Proceedings of the Copper 2016.

[18] Arias L., Torres S., Toro C., Balladares E., Parra R., Loeza C., Villagrán C., Coelho P. (2018). Flash Smelting Copper Concentrates Spectral Emission Measurements. Sensors.

[19] Sun Y., Lou C., Zhou H. (2011). A Simple Judgment Method of Gray Property of Flames Based on Spectral Analysis and the Two-Color Method for Measurements of Temperatures and Emissivity. Proc. Combust. Inst..

[20] Sun D., Lu G., Zhou H., Yan Y. (2011). Flame Stability Monitoring and Characterization through Digital Imaging and Spectral Analysis. Meas. Sci. Technol..

[21] Parada R. (2016). Combustión de Concentrados de Cobre en Hornos de Fusión Flash. Ph.D. Thesis.

[22] Gibson A.F. (1951). A Two-Colour Infra-Red Radiation Pyrometer. J. Sci. Instrum..

[23] Tuffrey N.E., Richards G.G., Brimacombe J.K. (1995). Two-Wavelength Pyrometry Study of the Combustion of Sulfide Minerals: Part II. Galena and Commercial Lead Concentrates. Metall. Mater. Trans. B.

[24] Gillard P., de Izarra C., Roux M. (2002). Study of the Radiation Emitted During the Combustion of Pyrotechnic Charges. Part II: Characterization by Fast Visualization and Spectroscopic Measurements. Propellants Explos. Pyrotech..

[25] Solnordal C.B., Jorgensen F.R.A., Koh P.T.L., Hunt A. (2006). CFD Modelling of the Flow and Reactions in the Olympic Dam Flash Furnace Smelter Reaction Shaft. Appl. Math. Model..

